# Is it still worth pursuing the repurposing of metformin as a cancer therapeutic?

**DOI:** 10.1038/s41416-023-02204-2

**Published:** 2023-02-23

**Authors:** Simon R. Lord, Adrian L. Harris

**Affiliations:** Department of Oncology, University of Oxford, Oxford, UK

**Keywords:** Drug development, Clinical trial design

## Abstract

Over the past 15 years, there has been great interest in the potential to repurpose the diabetes drug, metformin, as a cancer treatment. However, despite considerable efforts being made to investigate its efficacy in a number of large randomised clinical trials in different tumour types, results have been disappointing to date. This perspective article summarises how interest initially developed in the oncological potential of metformin and the diverse clinical programme of work to date including our contribution to establishing the intra-tumoral pharmacodynamic effects of metformin in the clinic. We also discuss the lessons that can be learnt from this experience and whether a further clinical investigation of metformin in cancer is warranted.

## Introduction

A drug is described as being ‘repurposed’ when it exhibits clinical benefit for the treatment of cancer patients, despite being initially developed for an unrelated indication. As drug development costs rise for new entities, there is a growing attraction in looking to off-patent medicines that have established safety and pharmacokinetic profiles potentially reducing the time to entry into the oncology clinic. Indeed, a recent estimate suggested that the median cost to develop a single cancer drug is $648 million [1], although other analyses have suggested far higher sums [2]. This outlay directly results in the great expense of drug therapy for patients and health systems, often more than $100,000 per year for new cancer drugs, and contributes to inequality of access to cancer treatment. For rare cancers, where low commercial returns may be prohibitive for drug development, repurposing may be of particular value.

Metformin is the most widely prescribed medicine for type 2 diabetes worldwide and on the World Health Organisation’s list of essential medicines. A series of epidemiological studies which suggested that metformin may reduce cancer incidence in diabetic populations sparked great interest in its potential as a cancer treatment. However, there remains some debate as to its pharmacodynamic effects in tumours and recent randomised trials have not clearly demonstrated clinical benefit for any cancer indication to date. In this perspective article, we present a summary of the history of preclinical and clinical studies that informed the repurposing of metformin, discuss how this programme of work could have been better focused and coordinated and lastly describe oncological indications where there remains a strong rationale for investigation.

## Metformin’s mechanism of action

As has typically been the case drug repurposing programmes, the interest in metformin was serendipitous. Two decades ago, researchers trying to understand the metabolic effects of metformin in diabetic patients discovered that it inhibits Complex 1 of the mitochondrial respiratory transport chain, the consequence of which was the activation of the cellular regulator of energy homoeostasis, AMP-activated protein kinase (AMPK) [3, 4]. AMPK is known to be a tumour suppressor that regulates a number of downstream anabolic pathways critical to tumour cell proliferation. On this basis, a pilot epidemiological case–control study was carried out and the analysis suggested that patients with Type 2 Diabetes Mellitus on metformin were less likely to develop cancer compared to patients on other diabetes drugs [5]. This finding led to a host of preclinical studies, which suggested that under laboratory conditions and with doses substantially greater than peak plasma level in patients, metformin possessed a number of anti-cancer properties including synergy with cytotoxic chemotherapy and radiotherapy.

Despite great effort and multiple published preclinical studies, the actual mechanism of action of metformin in tumour cells remains a topic of debate. As described above it is clear, at least when cells are treated with high doses, that metformin inhibits Complex-1 activity and cellular respiration. This was demonstrated in a series of elegant experiments in which metformin-resistant *Saccharomyces cerevisiae* NADH dehydrogenase NDI1 was overexpressed. In the same study the administration of metformin to mice inhibited the growth of control xenografts but not those expressing NDI1 [6]. In models, the consequences of inhibiting Complex 1 and hence the tricarboxylic acid cycle has been shown to check the funnelling of carbon from glucose to the anabolic building blocks needed for cell proliferation [7]. However, in patients, it remains unclear as to the degree to which the disruption of carbon metabolism or induction of energy stress and subsequent AMPK activation might be most critical to any antiproliferative effects. The further preclinical investigation has suggested a host of pleiotropic effects of metformin in cancer cells but it seems likely that many if not all of these are downstream consequences of metformin inhibiting complex 1 and subsequent AMPK activation, rather than the drug engaging multiple targets. For example, metformin has been shown to inhibit AKT/mTOR signalling and suppress fatty acid synthesis in an AMPK-dependent manner [8–10]. Metformin-induced AMPK activation has also been shown to reduce cancer cell proliferation through several other mechanisms, including activation of cMYC, HIF-1α and DICER1 [11].

However, an alternative hypothesis focussed on metformin’s effects on systemic ‘host’ metabolism has been proposed. Within the liver, activation of AMPK has been shown to reduce gluconeogenic gene expression in hepatocytes [12], increase insulin receptor activity and enhance translocation of glucose transporters [13]. It has also been proposed that biguanides inhibit glucagon signalling in the liver in an AMPK-independent manner, possibly by increasing AMP levels secondary to inhibition of Complex 1. AMP then by binding to adenylate cyclase downregulates cAMP-PKA activity suppressing gluconeogenesis [14]. Activation of the insulin receptor promotes downstream PI3K-AKT-mtor signalling and growth in tumour models [15] and increased insulin levels are associated with higher cancer incidence and mortality [16]. Hence, by reducing circulating insulin and glucose levels it is postulated that metformin may reduce insulin-mediated tumorigenesis and cancer progression, perhaps most relevant to patients with metabolic syndrome or type 2 diabetes although metformin has been shown to reduce insulin levels in cancer patients without these conditions [17].

## Pharmacodynamic clinical studies

In the clinic, metformin’s anti-cancer effects were initially evaluated in several small pharmacodynamic clinical trials. These typically used ‘window of opportunity’ designs often prior to surgery and compared assays using the diagnostic and surgical tumour sample with a course of metformin in between to assess the drug’s effects on cancer biology. The endpoints and findings of these studies are described in Table 1 but in summary almost all of these early studies designated the well-validated marker of proliferation, Ki67, as the primary endpoint. Other immunohistochemical markers were often assayed, in particular for immunohistochemical markers of apoptosis, AMPK and mTOR pathway activation, however detailed characterisation of metformin’s effects on tumour biology was not evaluated. To address this, we undertook a radiogenomic ‘window-of-opportunity’ study in 40 non-diabetic patients with primary breast cancer, linking FDG–PET tumour uptake to tumour transcriptomic and metabolomic profiling. Here, we observed the upregulation of several transcriptomic pathways linked to mitochondrial metabolism and change in the levels of a number of mitochondrial metabolites suggesting that metformin disrupted mitochondrial metabolism at clinical dosing. A reactive increase in mitochondrial oxidative phosphorylation gene transcription linked to metformin resistance and two distinct metabolic responses in breast cancer were observed. Furthermore, we showed that metformin increases 18-FDG flux in primary breast cancer concomitant to the increased expression of multiple genes regulating glycolysis, glucose transport and glutamine metabolism. This was consistent with breast tumours upregulating well-described mitochondrial metabolic resistance pathways in response to metformin adding weight to the potential of previously proposed strategies to target these pathways, in combination with metformin [17, 18].

**Table 1:** Window of opportunity pharmacodynamic clinical studies evaluating metformin’s effects on tumour biology.

Author/year	Tumour type	Design/sample size	Primary tumour assays	Summary
Hadad et al. [56]; Hadad et al. [57]	Breast cancer	RCT *n* = 55, 32 (MET) vs. 23 (CT)	IHC Ki67 Caspase 3, pAMPK	1. Reduced proliferation 2. Reduced apoptosis 3. Activation of AMPK
Bonnani et al. [58]; Cazzaniga et al. [59]	Breast cancer	RCT *n* = 200, 100 (MET) vs. 100 (placebo CT)	IHC Ki67 TUNEL	1. Reduced proliferation in subgroups with HOMA-IR score >2.8 2. No change in apoptosis
Niraula et al. [60]; Dowling et al. [61]	Breast cancer	Single arm *n* = 39	IHC Ki67 TUNEL IR pAMPK, pACC	1. Reduced proliferation 2. Increased apoptosis 3. Higher baseline glucose levels associated with more overt decrease Ki67 4. Reduced IR expression 5. No activation of AMPK
Kalinsky et al. [62]	Breast cancer with BMI ≥25 kg/m	Single arm (historical controls) *n* = 35	IHC Ki67	No change in proliferation
Lord et al. [17, 18]	Breast cancer	Single arm *n* = 40	FDG–PET-CT RNASeq Metabolomics	1. Increase FDG flux consistent with increased tumour glucose uptake 2. Increased expression of multiple mitochondrial pathways 3. Decreased levels of some mitochondrial metabolites 4. Two metabolic response patterns linked to the differential change in proliferation
Laskov et al. [63]	Endometrial cancer	Single arm (historical controls) *n* = 11	IHC Ki67 pAMPK pS6	1. Reduced proliferation 2. No activation of AMPK 3. Reduced mTOR signalling
Mitsuhashi et al. [64]	Endometrial cancer	Single arm (historical controls) *n* = 31	IHC Ki67 pAMPK pS6, pERK	1. Reduced proliferation 2. Activation of AMPK 3. Reduced mTOR signalling
Schuler et al. [65]	Endometrial cancer	Single arm *n* = 20	IHC Ki67 pAMPK pS6, pAKT, p4EBP1 ER	1. Reduced proliferation 2. No activation of AMPK 3. Reduced mTOR signalling 4. Reduced ER expression
Sivalingam et al. [66]	Endometrial cancer	Not randomised *n* = 40, 28 (MET) vs. 12 (CT)	IHC Ki67 pS6, pAKT, p4EBP1, pACC p-ACC, p-S6, p4EBP1, ER, PgR	1. No change in proliferation 2. Changes in expression of markers of mTOR signalling in both MET and CT groups 3. Reduced ER and PR expression
Kitson et al. [67]	Endometrial cancer	RCT *n* = 88, 45 (MET) vs. 43 (placebo CT)	IHC Ki67 pS6, pAKT, p4EBP1	1. No change in proliferation 2. No change in mTOR signalling
Petchsila et al. [68]	Endometrial cancer	RCT *n* = 49, 25 (MET) vs. 24 (placebo CT)	IHC Ki67	Reduced proliferation
Joshua, 2014 [69]	Prostate cancer	Single arm *n* = 22	IHC Ki67 pAMPK p4EBP1	1. Reduced proliferation 2. No activation of AMPK 3. Reduced mTOR signalling
Nguyen et al. [70]	Prostate cancer	RCT *n* = 20, 10 (MET) vs. 10 (placebo CT)	Metformin concentration IHC Ki67 Cyclin D1 CC3 pS6	1. Metformin distributes to prostate tissue 2. No change in proliferation 3. No change in apoptosis 4. No change in cell cycle regulation 5. No change in mTOR signalling
Brown et al. [71]	Ovarian cancer	Single arm (historical controls) *n* = 38	Flow cytometry	Reduction in ALDH^+^CD133^+^ ovarian cancer stem cell population
Curry et al. [72]	Head and neck cancer	Single arm *n* = 50	Mass spectroscopy imaging IHC Ki67 TUNEL CAV1, GALB, MCT4	1. Increase in lactate levels 2. No change in proliferation 3. No change in apoptosis 4. Change in expression of markers of glycolytic metabolism
Han et al. [42]	Cervical cancer	RCT *n* = 13, 10 (MET) vs. 3 (CT)	FAZA PET-CT	Reduction in cervical tumour hypoxia

*RCT* randomised controlled trial, *MET* metformin, *CT* control, *IHC* immunohistochemistry, *ER* oestrogen receptor, *PgR* progesterone receptor, *HOMA-IR* homoeostatic model assessment for insulin resistance, *PET-CT* positron emission tomography-computed tomography, *FDG* fluorodeoxyglucose, *FAZA* fluoroazomycin arabinoside.

## Efficacy studies

Results have now been presented from a number of randomised trials in different settings which in most cases have not demonstrated clinical benefit for metformin as a cancer treatment. These studies have assessed the combination of metformin with chemotherapy, endocrine and other targeted therapy in a number of different tumour types as summarised in Table 2. Most notably, the MA.32 study was a Phase III randomised trial that recruited over 3600 patients with high-risk operable breast cancer randomised to 850 mg metformin or placebo for 5 years. The investigators concluded that the addition of adjuvant metformin did not lead to an improvement in disease-free survival for either oestrogen receptor-positive or negative breast cancer [19]. An exploratory analysis did suggest that there might be some benefit in patients with HER2-positive disease and who genotyped for the C allele of the rs11212617 single-nucleotide polymorphism although the authors concluded that this would need to be confirmed with further prospective study [19, 20]. Tumour hypoxia is strongly linked to radiotherapy resistance and metformin has been shown to improve tumour oxygenation and radiotherapy response in xenograft models [21]. Hence, the combination of metformin and chemoradiotherapy has been investigated in patients with non-small lung cancer in two randomised trials but again with no evidence of benefit [22, 23]. A handful of studies have had encouraging results but with insufficient sample sizes to be firmly conclusive [24–28].

**Table 2:** Randomised efficacy trials published to date evaluating the clinical benefit of metformin as a primary endpoint.

Trial short title or study author	Tumour type	Design/sample size	Primary outcome measure	Summary
MA.32 [19]	Early breast cancer	RCT (1:1) Adjuvant metformin or placebo *n* = 3643 (2533 ER/PgR+ve breast cancer)	DFS in ER/PgR+ve breast cancer	1. No difference in DFS for ER/PgR+ve BC 2. Futility declared after interim analysis for patients who were ER/PgR-ve
METTEN [73]	Early HER2+ve breast cancer	RCT (1:1) Neoadjuvant Paclitaxel-FEC and trastuzumab +/− metformin *n* = 144	pCR rate	No difference in pCR response rate
I-SPY2 [28]	Early HER2-ve breast cancer	RCT (multi-arm study) Neoadjuvant Paclitaxel-AC +/− ganitumumab and metformin *n* = 234	pCR rate	Increase in pathological complete response rate but did not meet prespecified threshold for further investigation
El-Haggar et al. [26]	Early HER2-ve breast cancer	RCT (1:1) Adjuvant systemic therapy +/− metformin *n* = 129	DFS	Improvement in DFS for metformin arm
MYME [74]	Advanced HER2-ve breast cancer	RCT (1:1) Liposomal doxorubicin +/− metformin *n* = 122	PFS	No difference in PFS
Zhao et al. [75]	Advanced ER+ve HER2-ve breast cancer	RCT (1:1) Aromatase inhibitor +/− metformin *n* = 60	PFS	No difference in progression-free survival
Pimentel et al. [76]	Advanced breast cancer	RCT (1:1) Physician’s choice of chemotherapy +/− metformin *n* = 40 Trial stopped early due to slow accrual	PFS	No difference in progression-free survival
TAXOMET [77]	Advanced prostate cancer	RCT (1:1) Docetaxel +/− metformin *n* = 99	PSA response rate	No difference in PSA response rate
MANSMED [24]	Advanced prostate cancer	RCT (1:1) Androgen deprivation therapy +/− metformin *n* = 144	CRPC-FS	Improvement in CRPC-FS for metformin arm
Zheng et al. [78]	Epithelial ovarian cancer	RCT (1:1) Paclitaxel and carboplatin +/− metformin *n* = 44	PFS	No difference in PFS
GOG-0286B [79]	Stage III-IVB endometrial cancer	RCT (1:1) Paclitaxel and carboplatin +/− metformin *n* = 469	OS	No difference in OS
OCOG-ALMERA [22]	Non-small cell lung cancer	RCT (1:1) Chemoradiotherapy +/− metformin *n* = 54 Trial stopped early due to slow accrual	Proportion of patients who experienced a treatment failure event within 12 months	Metformin associated with worse outcome
NRG-LU001 [23]	Locally advanced non-small cell lung cancer	RCT (1:1) Chemoradiotherapy and consolidation radiotherapy +/− metformin *n* = 170	PFS at 1 year	No difference in PFS
Li et al. [80]	Advanced non-small cell EGFR mutant lung cancer	RCT (1:1) Gefitinib +/− metformin *n* = 224	PFS at 1 year	No difference in PFS
Arrieta et al. [25]	Advanced non-small cell EGFR mutant lung cancer	RCT (1:1) Physician’s choice of TKI +/− metformin *n* = 139	PFS	Improvement in PFS for metformin arm
Lee et al. [81]	Advanced non-small cell EGFR-ALK wild-type lung cancer	RCT (1:1) Carboplatin and gemcitabine +/− metformin *n* = 164	PFS	No difference in PFS
Marrone et al. [27]	Advanced non-small lung adenocarcinoma	RCT (3:1) Carboplatin, paclitaxel and bevacizumab +/− metformin *n* = 25 Trial stopped early due to slow accrual	PFS at 1 year	Improvement in PFS for metformin arm
GEM [82]	Advanced pancreatic cancer	RCT (1:1) Gemcitabine and erlotinib +/− metformin *n* = 121	Overall survival	No difference in overall survival
PACT-17 [83]	Advanced pancreatic cancer	RCT (1:1) Cisplatin, epirubicin, capecitabine and gemcitabine +/− metformin *n* = 60	PFS at 6 months	No difference in PFS

*RCT* randomised controlled trial, *ER* oestrogen receptor, *PgR* progesterone receptor, *DFS* invasive disease-free survival, *pCR* pathological complete response rate, *PFS* progression-free survival, *CRPC-FS* Castration-resistant prostate cancer-free survival, *OS* overall survival.

## Can lessons be learnt from the metformin experience?

When the MA.32 study commenced recruitment in 2010 there was very limited information regarding the pharmacodynamic effects of metformin in breast cancer at therapeutic doses from the clinic. This impacted on the ability to develop rationale clinical trial designs that took into account markers of selection, resistance, treatment combination and dosing. As has been frequently been demonstrated in drug development without appropriate patient selection clinical benefit may be masked.

Initially, there was little effort to establish the tolerability and possible advantage of higher dose levels of metformin in the context of treatment for cancer where a greater risk/side effect profile might be acceptable. Efforts have now been made to evaluate different dose levels for metformin and its biguanide cousin phenformin in various therapeutic combinations [29–31]. However, to our knowledge, a well-designed dose escalation study of metformin with detailed tumour pharmacodynamic assessment is still awaited.

An example of a well-structured programme of work that could be taken as template to repurpose an anti-mitochondrial agent for cancer therapy is the ongoing evaluation of the anti-parasitic drug atorvaquone as a radio-sensitiser. A decade ago, a group of investigators carried out a high throughput screen for drugs that reduced oxygen consumption and hence, potentially tumour hypoxia. Atovaquone is an anti-malarial agent and ubiquinone analogue that inhibits mitochondrial complex III and was identified as a ‘top hit’ in this screen. In vivo, atovaquone reduced tumour hypoxia and sensitised xenograft models to radiotherapy [32]. To determine whether atovaquone could reduce tumour hypoxia in patients, a pharmacodynamic clinical study compared 15 atovaquone treated versus 15 untreated non-small cell lung cancer (NSCLC) patients, recruited sequentially. Here, [18F]-fluoromisonidazole (FMISO) PET-CT demonstrated a significant reduction in hypoxia in the atovaquone group and this was corroborated using a transcriptomic hypoxia gene expression signature [33]. An ongoing dose escalation study, the ‘ARCADIAN trial’ is designed to ascertain the recommended phase 2 dose of atovaquone in combination with concurrent chemoradiotherapy in locally advanced NSCLC.

The contrast here with the approach to metformin is clear. The work was led by a team of collaborators who worked together throughout each stage of the drug development project to ensure that each step was informed by the prior. At an early point during clinical evaluation detailed pharmacodynamic assessment of the drug was carried out at several dose levels. We believe this stepwise approach to pharmacodynamic characterisation prior to Phase II/III efficacy trials optimises the chances of success in a drug repurposing programme.

## Future directions: is further clinical investigation of metformin in cancer warranted?

Given the results of the randomised efficacy trials enthusiasm to develop further clinical studies of metformin as a treatment for established cancers is waning. However, preclinical and clinical pharmacodynamic data obtained since the design of early clinical efficacy studies has now informed new avenues of investigation.

Markers are now being established that may define response to Complex-1 inhibitors such as mutations in the SWI-SNF complex [34]. Mitochondrial mutations in genes encoding for Complex 1 have also been proposed as markers of sensitivity for biguanide therapy [35] although mitochondrial heteroplasmy and the dynamic negative and positive enrichment of mitochondrial mutations may prevent their application as biomarkers. The transcription factor STAT3 is frequently activated in a variety of malignancies and emerging data points toward STAT3-mediated upregulation of OXPHOS as a mechanism of survival in drug-resistant tumours and a potential marker for drugs targeting mitochondrial metabolism [36–38]. The biobanking of translational samples from the trials already carried out to date may facilitate exploratory research to evaluate some of these emerging markers of susceptibility to anti-mitochondrial therapy with the opportunity for future trials with appropriate stratification. We suggest ‘window’ studies over short time frames for selected tumours may allow stratification of patients by evaluating dynamic response and highlight additional drug combination opportunities. If these had been performed a priori for metformin it may have aided trial design and outcome.

A number of animal and human studies have shown that metformin can alter the metabolism of gut microbiota [39, 40]. Transfer of faeces from obese mice treated with metformin into untreated mice inhibited tumour growth independently of changes in body mass, blood glucose or serum insulin. The study authors proposed that metformin treatment led to a proportionate increase in short-chain fatty acid-producing microbes and faecal transfer then led to reprogramming of tumour metabolism specifically changes in lipid homoeostasis [41]. To date, these approaches have been unexplored in the clinic.

Metformin, by inhibiting oxidative respiration and hence oxygen consumption has been shown to reduce hypoxia in tumour models [6] and more recently in a clinical study of patients with advanced cervical cancer using fluoroazomycin arabinoside (FAZA) PET-CT [42]. Via a number of mechanisms, hypoxia has been shown to suppress the anti-tumour immune response and this may be a significant mechanism of resistance to immune checkpoint immunotherapy [43]. Preclinical data have suggested that by remodelling the hypoxic tumour microenvironment metformin could potentiate the effect of anti PD-1 immunotherapy [44]. Metformin may enhance tumour immunosurveillance in ways other than reducing hypoxia in the tumour microenvironment. AMPK activation in immune cells leads to phosphorylation of PD-L1, subsequent PD-L1 glycosylation and its accumulation in the endoplasmic reticulum and degradation [45]. In syngeneic in vivo cancer models metformin enhanced the anti-tumour effect of anti-CTLA-4 therapy [45]. In another model metformin-induced AMPK activation was shown to inhibit PD-1 gene expression in CD8 +  T lymphocytes and in metformin-treated lung cancer patients there was an increase in the frequency of memory stem and central memory T cells [46]. Metformin-induced AMPK activation may downregulate CD39 and CD79 gene expression thereby reducing myeloid-derived suppressor cell-driven immunosuppression [47]. Tumour-associated macrophages have been shown to be immunosuppressive through production of specific immunomodulatory cytokines promoting tumour growth. The preclinical investigation has shown that metformin can alter macrophage polarisation from an M2 to M1-like phenotype inhibiting tumour growth and angiogenesis and that this may be driven by activation of AMPK/ NF-κB signalling [48, 49].

Metformin and its role in cancer prevention is an area that has been underexplored in prospective studies. Indeed, the epidemiological data provide a strong rationale for testing this hypothesis in selected groups of patients, for example, obese or insulin-resistant individuals and now early clinical trial data is emerging in support. One clinical study investigated metformin’s potential utility in preventing tamoxifen-induced endometrial hyperplasia showing reduced endometrial thickness on transvaginal ultrasound for metformin-treated patients compared to the placebo control group [50]. Metformin has been shown to suppress intestinal polyp growth in a murine model of familial adenomatous polyposis coli [51] and a subsequent randomised clinical trial showed that metformin reduced the prevalence and number of metachronous adenomas or polyps after polypectomy following 12 months of treatment with metformin [52].

However, prevention studies designed to identify differences in cancer incidence are notoriously difficult to execute given the numbers of patients needed to properly power such a trial and the length of time it takes to complete adequate follow-up. However, opportunity lies in investigating the potential of metformin as cancer preventative for patients with cancer predisposition syndromes which will allow for smaller studies and shorter follow-up. For example, Li-Fraumeni syndrome (LFS) is a rare inherited cancer predisposition syndrome with a lifetime risk of cancer close to 100% by age 60 years in women and 73% in men. LFS is caused by germline pathogenic variants in the *TP53* tumour suppressor gene [53] and in studies of mice carrying a knock-in missense mutation of *TP53*, metformin increases their cancer-free survival [54, 55]. This has been attributed to metformin’s direct anti-mitochondrial effect, supported by clinical evidence of attenuated mitochondrial respiration in peripheral blood mononuclear cells (PBMCs) from metformin-treated *mTP53* carriers. On this basis, randomised clinical trials are now moving forward to evaluate whether metformin can reduce cancer incidence in this high-risk population.

In summary, outcomes from late-phase efficacy studies testing metformin as a repurposed cancer therapeutic have been disappointing. In a rush to establish its potential utility, such trials were designed prior to due diligence with regard to patient selection, mechanism of action and appropriate combination. New avenues of investigation in selected populations including the assessment of combination with immunotherapy, and potential as a cancer preventative agent still warrant well-designed clinical investigation.

## Data Availability

Not applicable.
